# Valproic acid exposure affects social visual lateralization and asymmetric gene expression in zebrafish larvae

**DOI:** 10.1038/s41598-024-54356-7

**Published:** 2024-02-23

**Authors:** Andrea Messina, Valeria Anna Sovrano, Greta Baratti, Alessia Musa, Alessandra Gobbo, Alice Adiletta, Paola Sgadò

**Affiliations:** Center for Mind/Brain Sciences, University of Trento, Piazza della Manifattura 1, 38068 Rovereto, TN Italy

**Keywords:** Autism spectrum disorders, Molecular neuroscience, Social behaviour

## Abstract

Cerebral asymmetry is critical for typical brain function and development; at the same time, altered brain lateralization seems to be associated with neuropsychiatric disorders. Zebrafish are increasingly emerging as model species to study brain lateralization, using asymmetric development of the habenula, a phylogenetically old brain structure associated with social and emotional processing, to investigate the relationship between brain asymmetry and social behavior. We exposed 5-h post-fertilization zebrafish embryos to valproic acid (VPA), a compound used to model the core signs of ASD in many vertebrate species, and assessed social interaction, visual lateralization and gene expression in the thalamus and the telencephalon. VPA-exposed zebrafish exhibit social deficits and a deconstruction of social visual laterality to the mirror. We also observe changes in the asymmetric expression of the epithalamic marker *leftover* and in the size of the dorsolateral part of the habenula in adult zebrafish. Our data indicate that VPA exposure neutralizes the animals’ visual field bias, with a complete loss of the left-eye use bias in front of their own mirror image, and alters brain asymmetric gene expression and morphology, opening new perspectives to investigate brain lateralization and its link to atypical social cognitive development.

## Introduction

Functional and structural lateralization has been documented in humans^1–3^ and many other vertebrate species^4,5^, including fish^6,7^. Human and animal model studies suggest a general pattern of specialization of the two hemispheres^4,8,9^: the right hemisphere seems to be specialized for social and emotional processing and response to danger and novelty^10–14^, while the left hemisphere attends to categorization and attention, and controls the fine motor skills that underly language dominance and the population-level right-handedness in humans and other animals^15,16^. Hemispheric dominance and functional lateralization seem to be critical for typical cognitive development^17^. Loss of cerebral lateralization (either weaker or absent asymmetry) underlies poorer cognitive abilities and, in some cases, is associated with brain disorders, including autism^18^.

Autism Spectrum disorder (ASD) comprises a heterogeneous group of conditions characterized by atypical social interaction and communication, restricted interests and repetitive behavior, and sensory processing abnormalities. A large body of literature suggests alterations in hemispheric functional asymmetry associated with ASD, emerging since early development^19^. Deficits in language processing^20–23^ and abnormal hemispheric activation in response to speech^24^ have been consistently described in ASD. Interestingly, atypical prevalence of handedness has also been observed in individuals with ASD^25^. In vertebrates with front-facing eyes and binocular vision, such as humans, lateralized processing can be documented and measured through the observation of visual field biases, as, for example, the strong left visual field bias demonstrated in humans in face detection^26,27^ and emotional processing (see for a review^28^). In addition to aberrant lateralized responses to speech and in language processing, lack of left visual field bias in face and emotional processing has been extensively reported in ASD^29,30^, suggesting altered functional and structural lateralization already at early developmental stages^31^. On the same line, neurophysiological and neuroimaging studies have indicated altered patterns of lateralized activation in cortical brain areas associated with configurational information and categorization of faces (e.g., fusiform face area)^32–36^.

In recent years, studies in vertebrate species displaying functional lateralization have significantly contributed to widening the knowledge about the mechanisms underlying brain asymmetry and its role in cognitive functions^4^. Zebrafish are increasingly emerging among the key model species to study functional and anatomical aspects of brain asymmetry^7^. Similar to the visual field biases measured in humans, thanks to positioning of the eyes laterally, one on each side of the body and an almost complete decussation of the optic chiasm, the perception and processing of stimuli in zebrafish can be inferred, already at early developmental stages, on the basis of the simple observation of eye dominance during spontaneous behavior^37^. Studies in zebrafish larvae suggest that social stimuli are processed by the right hemisphere, as revealed by a left visual field bias the larvae show while observing their image reflected in a mirror^38^. In addition to behavioral lateralization, the asymmetric development of the zebrafish epithalamus has been used as a model to study the relationship between brain asymmetry and behavior^39,40^. During the development of the zebrafish epithalamic region, the parapineal organ is located on the left side in most of the embryos, asymmetrically influencing the development of the habenular nuclei. As a consequence, the two dorsal habenula nuclei show differences in size, connectivity^41,42^ and in the expression of the *kctd12.1/leftover* gene, which is typically asymmetrically distributed, highly expressed in the left habenula compared to the right one^43^. Two other members of the same gene family, *kctd12.2/right-on* and *kctd8/dexter* have an opposite expression pattern^44^.

Given the fundamental contribution of cerebral asymmetry in brain organization and development, and its association with neuropsychiatric diseases, including autism, this study aimed to investigate brain lateralization in an animal model of ASD based on embryonic administration of valproic acid (VPA), a compound used to model the core signs of ASD in many vertebrate species^45–47^. VPA is an anticonvulsant known to interfere with development of the social brain, whose prenatal exposure is associated in humans with neural tube malformations, reduced cognitive function and an increased risk for developing ASD^48^. VPA embryonic exposure has been extensively used to model ASD core symptoms in diverse animal species^46^, including zebrafish^49^. Five-hour-post-fertilization (5 hpf) zebrafish larvae were exposed to one micromolar VPA for 24 and 48 h and assessed, at three- and four-weeks post-fertilization, for the social responses to their reflected image (mirror test) and to the conspecifics (social preference), respectively. At three-months-post-fertilization VPA-exposed zebrafish were assessed for asymmetric gene expression in the thalamus and in the telencephalon.

## Results

### Mirror test

**Figure 1:**
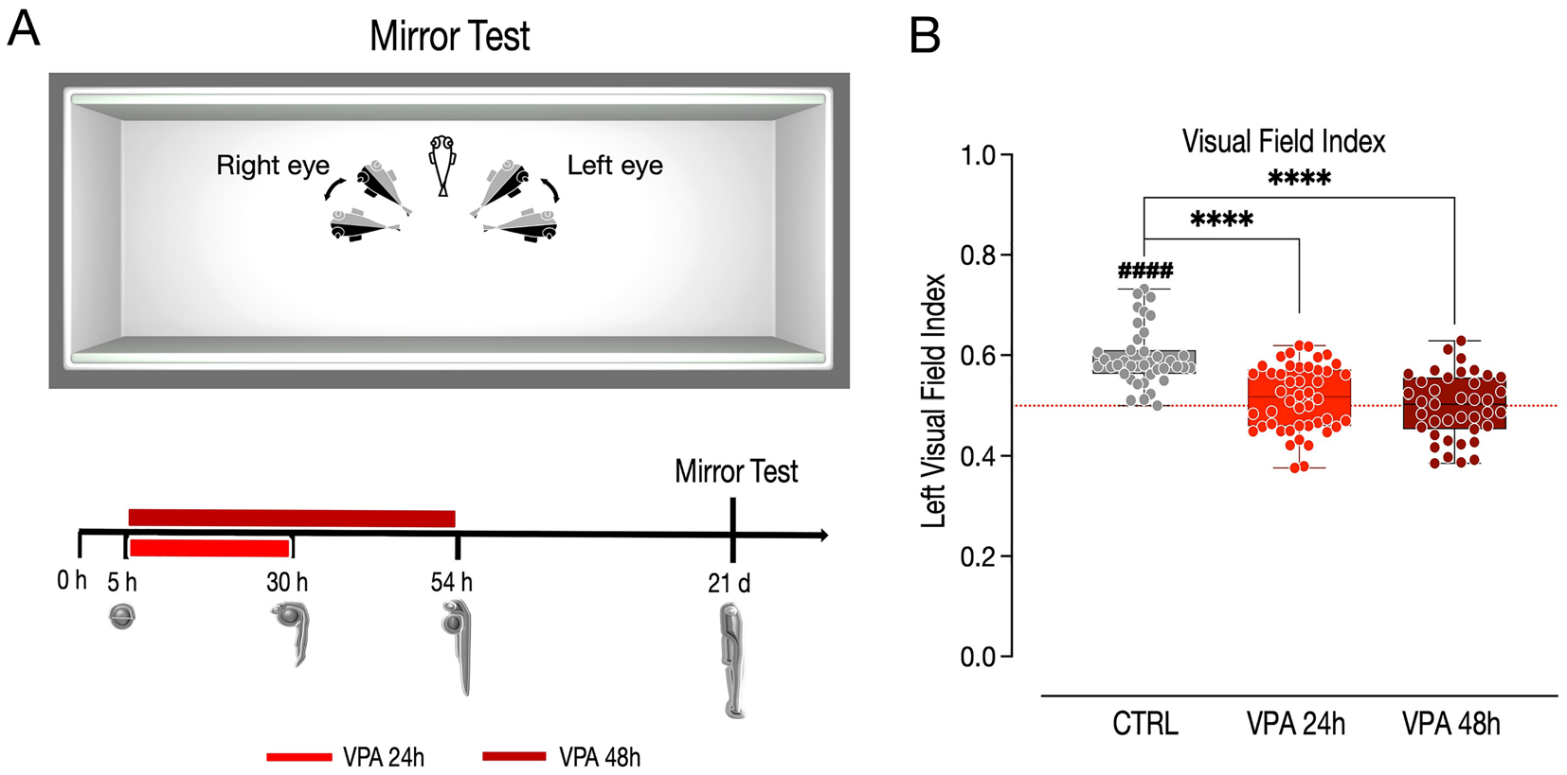
Mirror test. (**A**) Top, apparatus used for the mirror test, showing the position of the mirrors and the angles of viewing that defined monocular vision with the right or left eye. Data were discarded when the fish was perpendicular to the mirror (binocular stimulation, transparent fish) or when it formed an angle larger than 90º with respect to the closest mirror. Bottom, scheme of the experimental timeline, VPA treatment begin at 5 hpf and last for 24 or 48 h. The mirror test starts at 21 dpf. (**B**) Box and whisker plot (median, min to max) showing the visual field index. The number sign (#) indicate significant departures of the visual field index from chance level (0.5), marked by the red line. ^**####**^*p < *0.0001; ^****^p < 0.0001.

**Figure 2:**
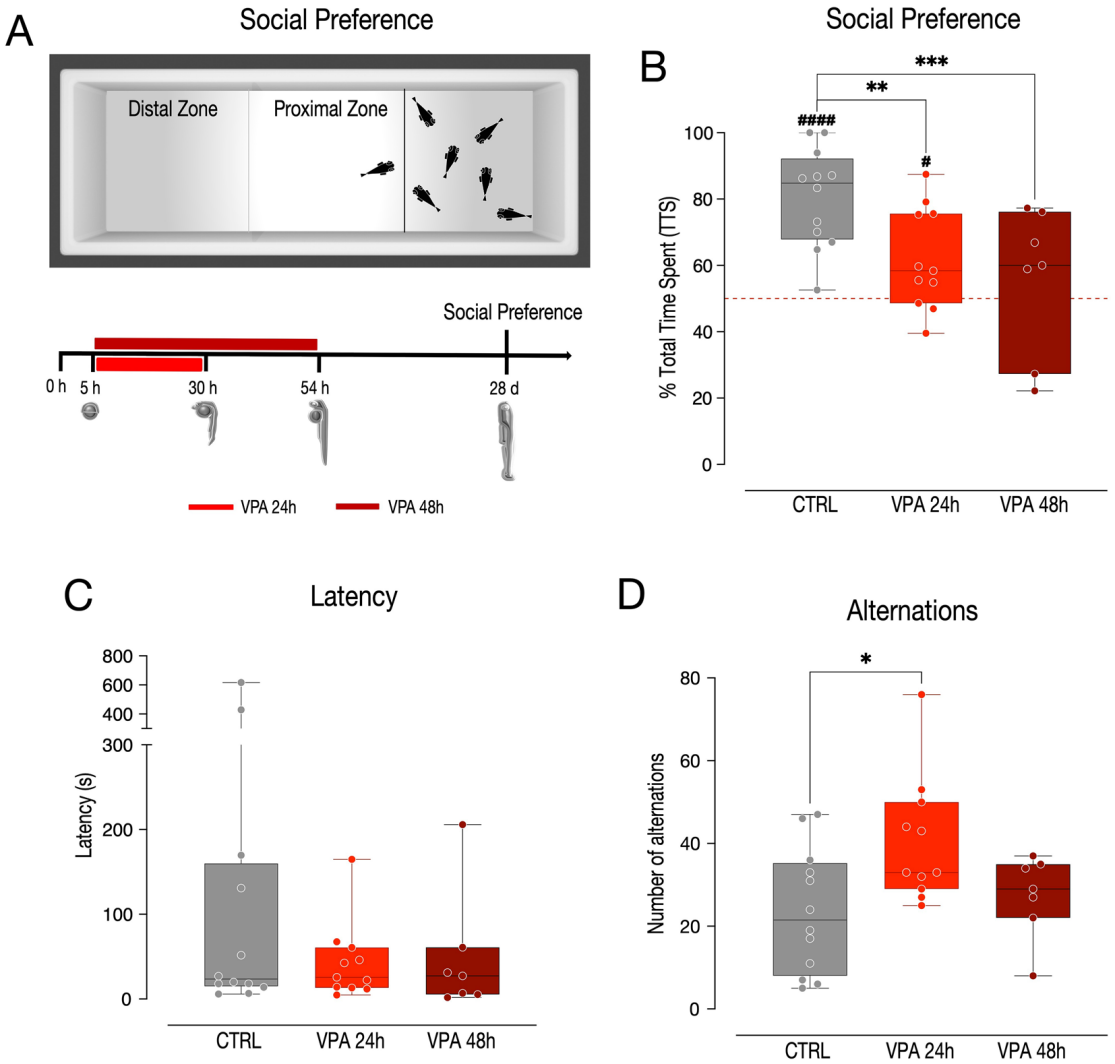
Social preference test. (**A**) Top, apparatus used for the social preference test, showing the conspecifics chamber and the two areas, the proximal and distal zone. Bottom, scheme of the experimental timeline, VPA treatment begin at 5 hpf and last for 24 or 48 h. The social preference test starts at 28 dpf. (**B**, **C**, **D**) Box and whisker plot (median, min to max) showing (**B**) the % of time spent in the proximal zone (social preference index), (**C**) the latency to change zone and (**D**) the number of alternations between proximal and distal zones. The number sign (#) indicate significant departures of the social preference index from chance level (50%), marked by the red line. ^**#**^*p < *0.05; ^**####**^*p < *0.0001; ^*^p < 0.05; ^**^p < 0.01; ^***^p < 0.001.

One hundred twenty-four larvae of the AB strain, treated with vehicle (39), 1 µM VPA for 24 (46) or 48 h (39), were subjected to the mirror test (Fig. 1A) to assess the effect of treatment on the left visual bias. The left visual bias was expressed as the frequency of left eye use when the fish were observing their reflection close to the mirror. The analysis of variance indicated a significant effect of treatment on the left visual field index (Fig. 1B; *F*_(2,121)_ = 27.76, *p < *0.0001), with a remarkable reduction, at the population level, of the use of the left eye during the test in both the VPA 24 and 48 h treatment group (Fig. 1B; pairwise comparisons: CTRL vs VPA 24 h *t*_(121)_ = 5.980, *p < *0.0001; CTRL vs. VPA 48 h *t*_(121)_ = 6.908, *p < *0.0001; VPA 24 h vs. VPA 48 h *t*_(121)_ = 1.206, *p =* 0.4518). In accordance with previous results, the vehicle-treated larvae displayed a stable bias for left eye use (Fig. 1B; one-sample *t*-test: CTRL t_(38)_ = 10.317, *p < *0.0001; group mean: CTRL 0.5960 [95% CI 0.5772–0.6148]), while both VPA treatment groups showed no preferential visual field use during the test (Fig. 2B; one-sample *t*-test: VPA 24 h *t*_(45)_ = 1.4861, *p =* 0.1442; VPA 48 h *t*_(38)_ = − 0.2105, *p =* 0.8344; group mean: VPA 24 h 0.5143 [95% CI 0.4949–0.5337]; VPA 48 h 0.4978 [95% CI 0.4771–0.5186]). This data suggests that VPA treatment impairs the behavioral lateralization shown by the larvae in response to their reflected image, as visual cues representing a conspecific.

### Social interaction test

To assess the effect of VPA on a more general sociability test, an independent group of larvae was subjected to the social interaction test. Thirty larvae of the AB strain treated with vehicle (12), 1 µM VPA for 24 h (11) or 48 h (7) underwent the social preference tests at four weeks of age (Fig. 2A). To account for potential habituation of the fish to the environment, the analysis of variance also evaluated the effect of time (three and five minutes, repeated measures) on the social preference and its interaction with VPA treatment. The results show no effect of time (*F*_(1,54)_ = 0.283, *p =* 0.597) nor of the interaction between time and treatment (*F*_(2,54)_ = 0.107, *p =* 0.899) but a significant effect of treatment (*F*_(2,54)_ = 11.813, *p < *0.0001) on the social preference index. Twenty-four and 48 h of treatment with VPA significantly decreased the preference of the fish to spend time in the chamber close to the conspecifics (Fig. 2B; pairwise comparisons: CTRL vs. VPA 24 h *t*_(54)_ = 3.791, *p =* 0.0011; CTRL vs. VPA 48 h *t*_(54)_ = 4.352, *p =* 0.0002; VPA 24 h vs. VPA 48 h *t*_(54)_ = 1.008, *p =* 0.5750). A very strong bias to remain in the compartment of the chamber adjacent to the conspecifics was detected in fish treated with vehicle (Fig. 2B; one-sample *t*-test CTRL t_(11)_ = 7.083, *p < *0.0001, group mean: 80.43% [95% CI 70.97%–89.88%]), the group of animals treated with VPA for 24 h showed a weaker, but significant, preference for the conspecifics (Fig. 2B; one-sample *t*-test VPA 24 h *t*_(10)_ = 2.592, *p =* 0.0269, group mean: 61.93% [95% CI 51.67%–72.18%]) while fish treated for 48 h did not show any preference for the proximal chamber (Fig. 2B; one-sample *t*-test VPA 48 h *t*_(6)_ = 0.659, *p =* 0.5343, group mean: 55.54% [95% CI 34.96%–76.12%]). To assess potential confounds on the social preference related to motor activity, the latency to the first change of zone and the number of alternations between the two zones during the social preference test were also evaluated. The statistical analysis showed no significant effect of treatment on the latency (Fig. 2C; *F*_(2,27)_ = 1.320, *p =* 0.2839), however a clear effect of treatment on the number of spontaneous alternations emerged (Fig. 2D; *F*_(2,27)_ = 4.385, *p =* 0.0224). Larvae treated with 1 µM VPA for 24 h, but not for 48 h, displayed a significant increase in the number of alternations between the proximal and the distal zone (Fig. 2D; pairwise comparisons: CTRL vs. VPA 24 h *t*_(27)_ = − 2.881, *p =* 0.0203; CTRL vs. VPA 48 h *t*_(27)_ =  − 0.586, *p =* 0.8288; VPA 24 h vs. VPA 48 h* t*_(27)_ = 1.911, *p =* 0.1550).

### Asymmetric gene expression analysis

**Figure 3:**
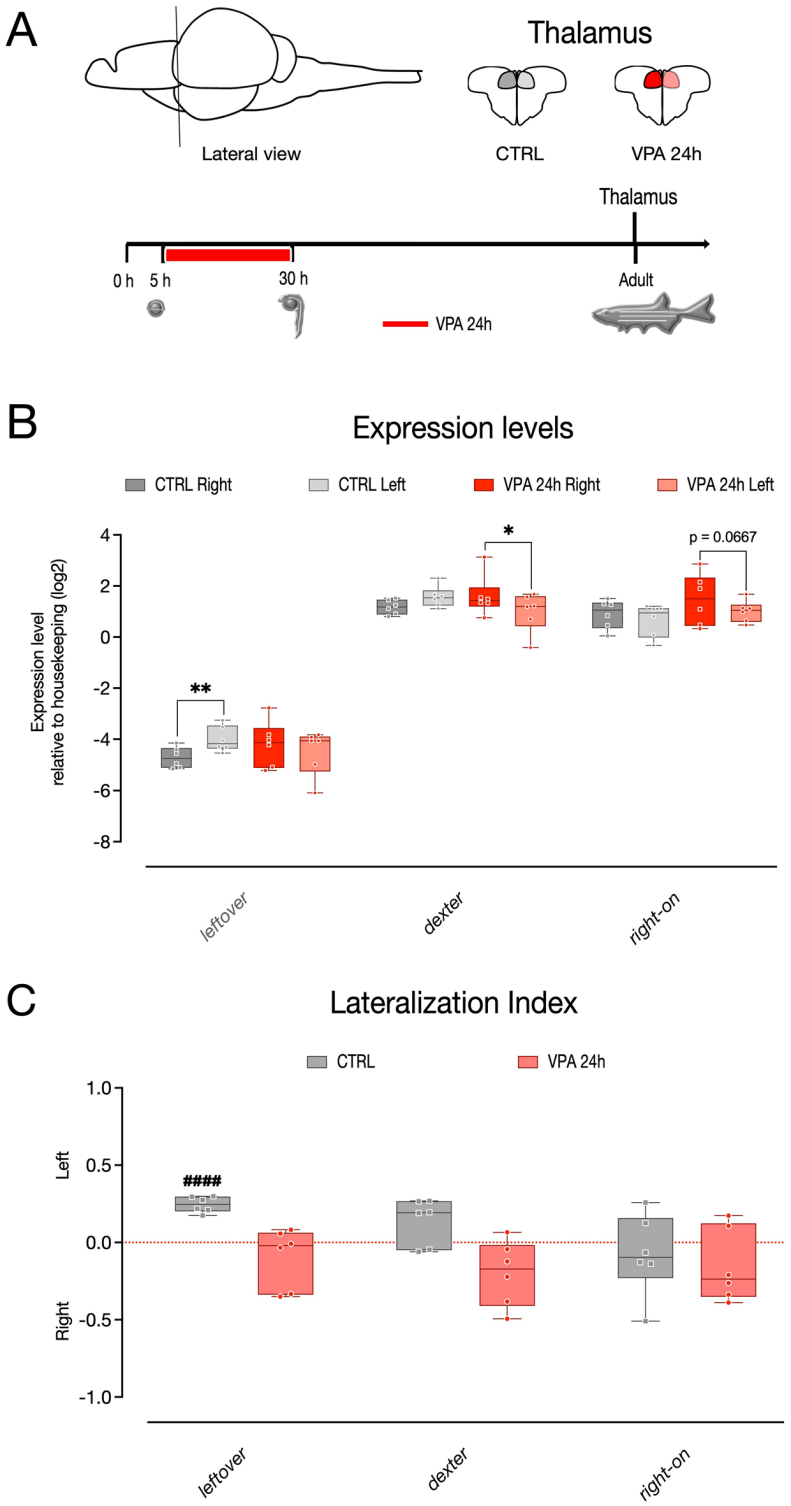
Gene expression in the left and right thalamus of zebrafish. (**A**) Top, schematic representation of thalamic regions selected for the analyses. Bottom, scheme of the experimental timeline, VPA treatment begin at 5 hpf and last for 24 h. Gene expression is analyzed in adult zebrafish. (**B**) Box and whisker plot (median, min to max) of relative expression (dCt, log2) values for each treatment group for *kctd12.1/lov*, *kctd8/dex* and *kctd12.2/ron* in the left and right thalamus of zebrafish. (**C**) Lateralization index for *kctd12.1/lov*, *kctd8/ dex* and *kctd12.2/ron*. The number sign (#) indicate significant departures of the lateralization index from chance level (0.5), marked by the red line. ^**####**^*p < *0.0001; ^*^p < 0.05; ^**^p < 0.01.

To investigate whether the functional lateralization disturbances mediated by VPA in the mirror test were also accompanied by alterations in brain asymmetry, the left and right thalami of three-month-post-fertilization (mpf) zebrafish were micro-dissected and the expression levels of the thalamic markers *kctd12.1/leftover* (*lov*), *kctd8/dexter* (*dex*) and *kctd12.2/right-on* (*ron*) evaluated (Fig. 3A; n = 6 animals per treatment group, 6 independent experiments). Considering that the behavioral analyses did not reveal substantial differences between zebrafish exposed to the same concentrations of VPA for 24 h or 48 h, gene expression was analyzed only in those larvae treated for 24 h. To assess the effect of treatment on the asymmetric expression of these genes, we fitted a linear mixed model, including treatment, brain side, and transcript type as fixed factors and the experimental unit (experiment) as random factor. We compared a model with random-intercepts only to one with random slopes and intercepts and found that the second model fitted the data significantly better. The statistical analysis indicated a significant difference in asymmetric gene expression in the treatment groups (interaction treatment * brain side *F*_(1,55)_ = 13.8159, *p =* 0.0005). We also found a difference in the expression levels of the three transcripts analyzed (main effect of transcript *F*_(2,55)_ = 1332.2072, *p < *0.0001) and no other significant main effects or interactions (main effect of treatment *F*_(1,55)_ = 0.2256, *p =* 0.6367; main effect of brain side *F*_(1,55)_ = 0.6442, *p =* 0.4257; treatment * transcript interaction *F*_(2,55)_ = 2.6251, *p =* 0.0815; brain side * transcript interaction *F*_(2,55)_ = 2.6440, *p =* 0.0801; treatment * brain side * transcript interaction *F*_(2,55)_ = 1.7248, *p =* 0.1877). The pairwise comparison between the levels of expression in the two hemispheres of each treatment groups revealed a higher expression of *kctd12.1/leftover* in the left hemisphere compared to right one, in the control samples (Fig. 3B and Table 1; *leftover* CTRL right–left: *t*_(55)_ = − 2.930, *p =* 0.0049), in line with previous reports^44,50,51^. This difference was however absent in zebrafish treated with VPA (Fig. 3B and Table 1; *leftover* VPA 24 h right–left: *t*_(55)_ = 1.195, *p =* 0.2374). No changes in the expression of *kctd12.2/right-on* was detected between the two hemispheres in the treatment groups (Fig. 3B and Table 1; *right-on* CTRL right–left: *t*_(55)_ = 0.980, *p =* 0.3313; *right-on* VPA 24 h right–left: *t*_(55)_ = 1.871, *p =* 0.0667), nor in the expression of *kctd8/dexter* in the control samples, while in VPA treated samples *kctd8/dexter* was expressed at lower levels in the left hemisphere compared to the right one (Fig. 3B and Table 1; *dexter* CTRL right–left: *t*_(55)_ = − 1.619, *p =* 0.1112; *dexter* VPA 24 h right–left: *t*_(55)_ = 2.470, *p =* 0.0166; see Table 1 for a summary of all pairwise comparisons). The analysis of the Lateralization index also indicated, as previously reported at different developmental stages^44,50,51^, that *kctd12.1/leftover* expression was asymmetric, predominant on the left side of the thalamus in the control group at this stage, as shown by the significant departure from chance levels of the lateralization index (Fig. 3C and Table 2; one sample t-test of left lateralization index *leftover* CTRL *t*_(5)_ = 11.73, *p < *0.0001). This lateralized expression was, however, absent in VPA-treated animals (Fig. 3C and Table 2; one sample t-test of left lateralization index *leftover* VPA 24 h *t*_(5)_ = 1.235, *p =* 0.2716). Differently from previous reports describing the asymmetric distribution of *kctd8/dexter* and *kctd12.2/right-on* primarily on the right hemisphere during development^44,50,51^, these thalamic markers were not clearly preferentially distributed in one of the hemispheres of the adult thalamus in any of the treatment groups (Fig. 3C and Table 2; one sample t-test of left lateralization index *dexter* CTRL t_(5)_ = 2.216, *p =* 0.0775; *dexter* VPA 24 h t_(5)_ = 2.326, *p =* 0.0675; *right-on* CTRL t_(5)_ = 0.7083, *p =* 0.5104; *right-on* VPA 24 h t_(5)_ = 1.587, *p =* 0.1773; see Table 2 for a summary of all one-sample t-test results).

**Table 1:** Gene expression analysis in the thalamus: pairwise comparison report.

Thalamus	CTRL						VPA					
	Dex		Lov		Ron		Dex		Lov		Ron	
	Right	Left	Right	Left	Right	Left	Right	Left	Right	Left	Right	Left
Mean	1.172	1.573	− 4.722	− 3.997	0.902	0.659	1.602	0.991	− 4.195	− 4.490	1.467	1.004
S.E.	0.194	0.194	0.194	0.194	0.194	0.194	0.335	0.335	0.335	0.335	0.335	0.335
N	6	6	6	6	6	6	6	6	6	6	6	6
Lower 95% CL	0.674	1.075	− 5.220	− 4.495	0.404	0.162	0.740	0.129	− 5.057	− 5.352	0.605	0.142
Upper 95% CL	1.670	2.070	− 4.225	− 3.500	1.400	1.157	2.464	1.853	− 3.333	− 3.628	2.329	1.867
Contrast (right–left)												
*t* ratio	− 1.619		− 2.930		0.980		2.470		1.195		1.871	
*p* value	0.111		**0.005**		0.331		**0.017**		0.237		0.067	
η^2^ (partial)^a^	− 0.935		− 1.692		0.566		1.426		0.690		1.080	
Lower 95% CL	− 2.419		− 3.176		− 0.918		− 0.058		− 0.794		− 0.404	
Upper 95% CI	0.549		− 0.208		2.050		2.910		2.174		2.564	

^a^Effect size calculated using the R package *emmeans*.

Significant values are in bold.

**Table 2:** Lateralisation Index in thalamus: one-sample t-test summary report.

Thalamus	CTRL			VPA		
	Dex	Lov	Ron	Dex	Lov	Ron
Mean	0.136	0.246	− 0.076	− 0.200	− 0.098	− 0.153
S.E.	0.061	0.021	0.108	0.086	0.079	0.097
N	6	6	6	6	6	6
Lower 95% CI	− 0.022	0.192	− 0.353	− 0.421	− 0.301	− 0.402
Upper 95% CI	0.293	0.299	0.200	0.021	0.106	0.095
*t* ratio	2.216	11.734	− 0.708	− 2.326	− 1.235	− 1.587
*p* value	0.078	**< 0.0001**	0.510	0.068	0.272	0.173
Cohen’s *d*^a^	0.90	4.79	− 0.29	− 0.95	− 0.50	− 0.65
Lower 95% CI	-0.09	1.82	− 1.09	− 1.90	− 1.34	− 1.51
Upper 95% CI	1.84	7.77	0.54	0.06	0.37	0.27

^a^Effect size calculated using the R package *effectsize*].

Significant values are in bold.

**Figure 4:**
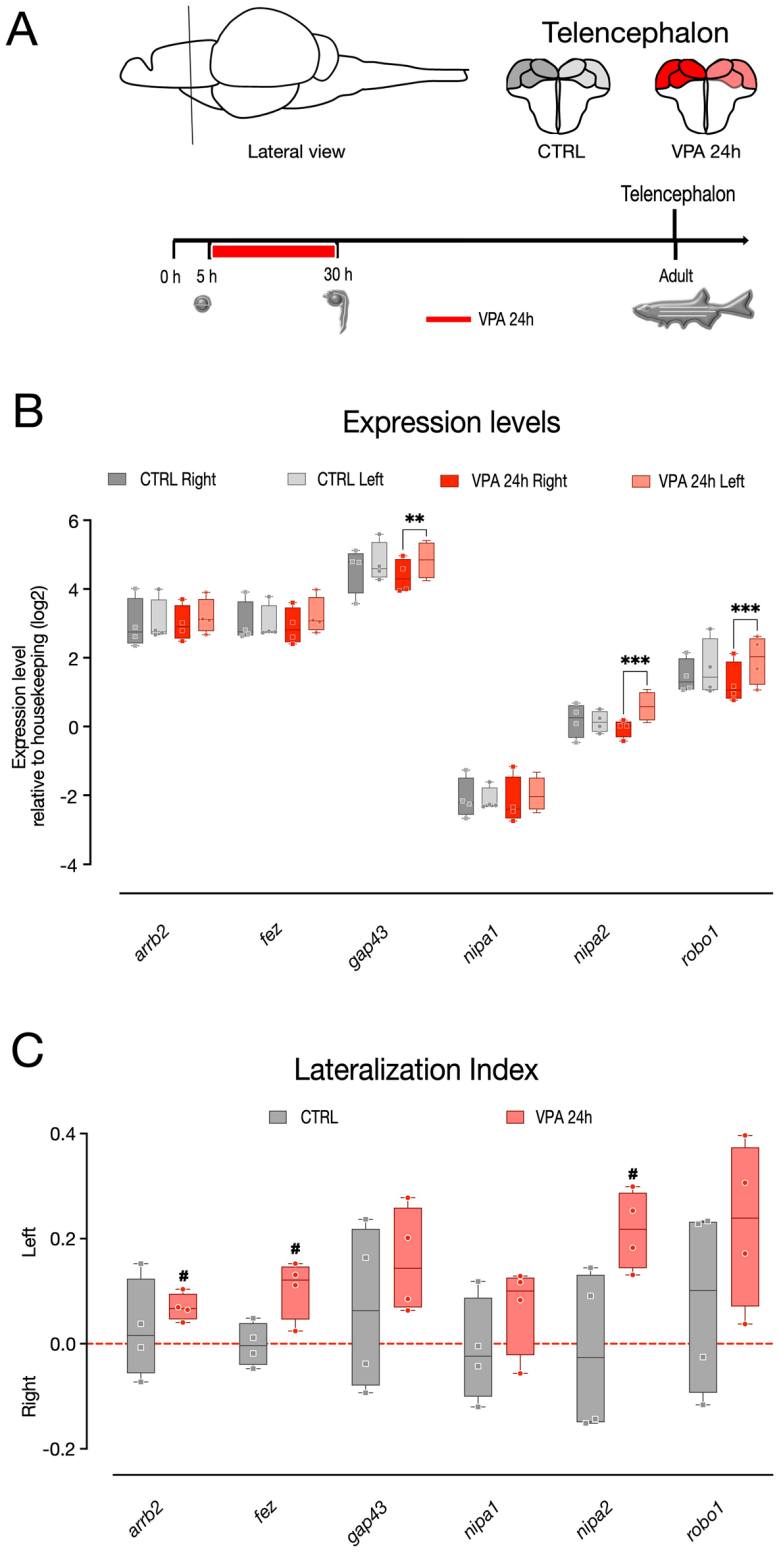
Gene expression in the left and right telencephalon of zebrafish. (**A**) Top, schematic representation of telencephalic (pallial and subpallial) regions selected for the analyses. Bottom, scheme of the experimental timeline, VPA treatment begin at 5 hpf and last for 24 h. Gene expression is analyzed in adult zebrafish. (**B**) Box and whisker plot (median, min to max) of relative expression (dCt, log2) values for each treatment group for *arrb2*, *fez, gap43, nipa1, nipa2* and *robo1* in the left and right telencephalon of zebrafish. (**C**) Lateralization index for *arrb2*, *fez, gap43, nipa1, nipa2* and *robo1*. The number sign (#) indicate significant departures of the left lateralization index from chance level (0.5), marked by the red line. ^**#**^*p < *0.05; ^**^p < 0.01; ^***^p < 0.001.

To assess brain asymmetry also in the telencephalon, the expression of pallial genes previously shown to be asymmetrically distributed in adult zebrafish^51^ was examined in VPA-exposed zebrafish. The left and right telencephala (including the pallium and the subpallium) were dissected, and then the expression of *arrb2, fez1, gap43, nipa1, nipa2* and *robo1* evaluated in the two hemispheres of 3 mpf zebrafish subjected to same treatment paradigm: 1 µM VPA at 5 hpf for 24 h (Fig. 4A; n = 4 animal per treatment group, 4 independent experiments). To assess the effect of treatment, brain side and transcript type on the expression levels, we again fitted a linear mixed model, including the experiment (experimental unit) as a random factor. Using a random slopes and intercepts model, the analysis revealed a significant asymmetric gene expression in the treatment groups (Fig. 4B; interaction treatment * brain side *F*_(1,69)_ = 8.5682, *p =* 0.0046) as well as a difference in the expression levels of the transcripts analyzed (main effect of transcript *F*_(5,69)_ = 1125.7494, *p < *0.0001) and an overall difference in the expression levels between hemispheres (main effect of brain side *F*_(1,69)_ = 17.3302, *p =* 0.0001). No other significant main effects or interactions emerged (main effect of treatment *F*_(1,69)_ = 0.0695, *p =* 0.7928; transcript * brain side interaction *F*_(5,69)_ = 1.0046, *p =* 0.4217; treatment * transcript interaction *F*_(5,69)_ = 0.1690, *p =* 0.9732; treatment * brain side * transcript interaction *F*_(5,66)_ = 0.4750, *p =* 0.7937). The pairwise comparison between the expression levels of the two hemispheres in the treatment groups indicated no difference in the control group for all the genes analyzed, differently from what was previously described^51^, while a significant difference between hemispheres was observed in the VPA treatment group for the gene *gap43*, *nipa2* and *robo1*, showing increased expression in the left hemisphere (Fig. 4B and Table 3; *gap43* VPA 24 h right–left: *t*_(69)_ = − 2.279, *p =* 0.0258; *nipa2* VPA 24 h right–left: *t*_(69)_ =  − 3.152, *p =* 0.0024; *robo1* VPA 24 h right–left: *t*_(69)_ =  − 3.377, *p =* 0.0012; see Table 3 for a summary of pairwise comparison results). Differently from previous reports^51^, the lateralization index for all the genes analyzed was not suggestive of lateralization in their distribution in the control groups however in some cases, the expression was asymmetrically distributed in the VPA-treated group, for example in the case of *arrb2*, *fez* and *nipa2*, for which significant departure from chance levels of the left lateralization indexes was observed (Fig. 4C and Table 4; one sample *t*-test of lateralization index *arrb2* VPA 24 h *t*_(3)_ = 5.336, *p =* 0.0129; *fez* VPA 24 h *t*_(3)_ = 3.272, *p =* 0.0336; *nipa2* VPA 24 h *t*_(3)_ = 5.818, *p =* 0.010; see Table 4 for a summary of all one-sample *t*-test results).

**Table 3:** Gene expression analysis in the telecephalon: pairwise comparison report.

Telencephalon	CTRL											
	arbb2		fez		gap43		nipa1		nipa2		robo1	
	right	left	right	left	right	left	right	left	right	left	right	left
Mean	2.966	3.047	3.013	3.009	4.565	4.763	− 2.083	− 2.118	0.182	0.138	1.452	1.688
S.E.	0.283	0.283	0.283	0.283	0.283	0.283	0.283	0.283	0.283	0.283	0.283	0.283
N	4	4	4	4	4	4	4	4	4	4	4	4
Lower 95% CL	2.064	2.145	2.112	2.108	3.664	3.862	− 2.984	− 3.019	− 0.720	− 0.763	0.550	0.787
Upper 95% CL	3.867	3.948	3.915	3.911	5.466	5.665	− 1.181	− 1.216	1.083	1.040	2.353	2.590
Contrast (right–left)												
*t* ratio	− 0.400		0.021		− 0.981		0.175		0.214		− 1.170	
*p* value	0.691		0.984		0.330		0.862		0.831		0.246	
η^2^ (partial)^a^	− 0.283		0.015		− 0.693		0.124		0.152		− 0.827	
Lower 95% CL	− 2.533		− 2.236		− 2.944		− 2.127		− 2.099		− 3.078	
Upper 95% CL	1.968		2.265		1.557		2.374		2.402		1.423	

Telencephalon	VPA											
	arbb2		fez		gap43		nipa1		nipa2		robo1	
	right	left	right	left	right	left	right	left	right	left	right	left
Mean	2.998	3.198	2.909	3.213	4.377	4.837	-2.172	-1.974	-0.050	0.588	1.254	1.937
S.E.	0.277	0.277	0.277	0.277	0.277	0.277	0.277	0.277	0.277	0.277	0.277	0.277
N	4	4	4	4	4	4	4	4	4	4	4	4
Lower 95% CL	2.116	2.317	2.028	2.332	3.496	3.956	− 3.053	− 2.855	− 0.931	− 0.294	0.373	1.056
Upper 95% CL	3.879	4.079	3.790	4.094	5.258	5.719	− 1.291	− 1.093	0.831	1.469	2.135	2.818
Contrast (right–left)												
*t* ratio	− 0.993		− 1.504		− 2.279		− 0.977		− 3.152		− 3.377	
*p* value	0.324		0.137		**0.026**		0.332		**0.002**		**0.001**	
η^2^ (partial)^a^	− 0.702		− 1.063		− 1.611		− 0.690		− 2.229		− 2.388	
Lower 95% CL	− 2.952		− 3.314		− 3.861		− 2.941		− 4.479		− 4.638	
Upper 95% CL	1.548		1.187		0.639		1.560		0.022		− 0.137	

^a^Effect size calculated using the R package *emmeans*.

Significant values are in bold.

**Table 4:** Lateralisation Index in telencephalon: one-sample *t*-test summary report.

Telencephalon	CTRL						VPA					
	arbb2	fez	gap43	nipa1	nipa2	robo1	arbb2	fez	gap43	nipa1	nipa2	robo1
Mean	0.028	− 0.001	0.067	− 0.012	− 0.015	0.080	0.069	0.105	0.157	0.068	0.217	0.228
S.E.	0.047	0.021	0.079	0.050	0.077	0.089	0.013	0.028	0.050	0.043	0.037	0.079
N	4	4	4	4	4	4	4	4	4	4	4	4
Lower 95% CI	− 0.123	− 0.067	− 0.184	− 0.171	− 0.261	− 0.203	0.028	0.015	− 0.003	− 0.068	0.098	− 0.022
Upper 95% CI	0.179	0.064	0.319	0.146	0.231	0.363	0.111	0.194	0.318	0.204	0.335	0.478
*t* ratio	0.586	− 0.070	0.851	− 0.246	− 0.191	0.899	5.336	3.728	3.116	1.596	5.818	2.904
*p* value	0.599	0.948	0.457	0.821	0.861	0.435	**0.013**	**0.034**	0.053	0.209	**0.010**	0.062
Cohen’s *d*^a^	0.29	− 0.04	0.43	− 0.12	− 0.10	0.45	2.67	1.86	1.56	0.80	2.91	1.45
Lower 95% CI	− 0.73	− 1.01	− 0.64	− 1.10	− 1.07	− 0.62	0.42	0.12	− 0.01	− 0.39	0.51	− 0.06
Upper 95% CI	1.28	0.95	1.43	0.87	0.89	1.46	4.90	3.55	3.06	1.91	5.31	2.89

^a^Effect size calculated using the R package *effectsize*].

Significant values are in bold.

### Habenula neuroanatomical analysis

**Figure 5:**
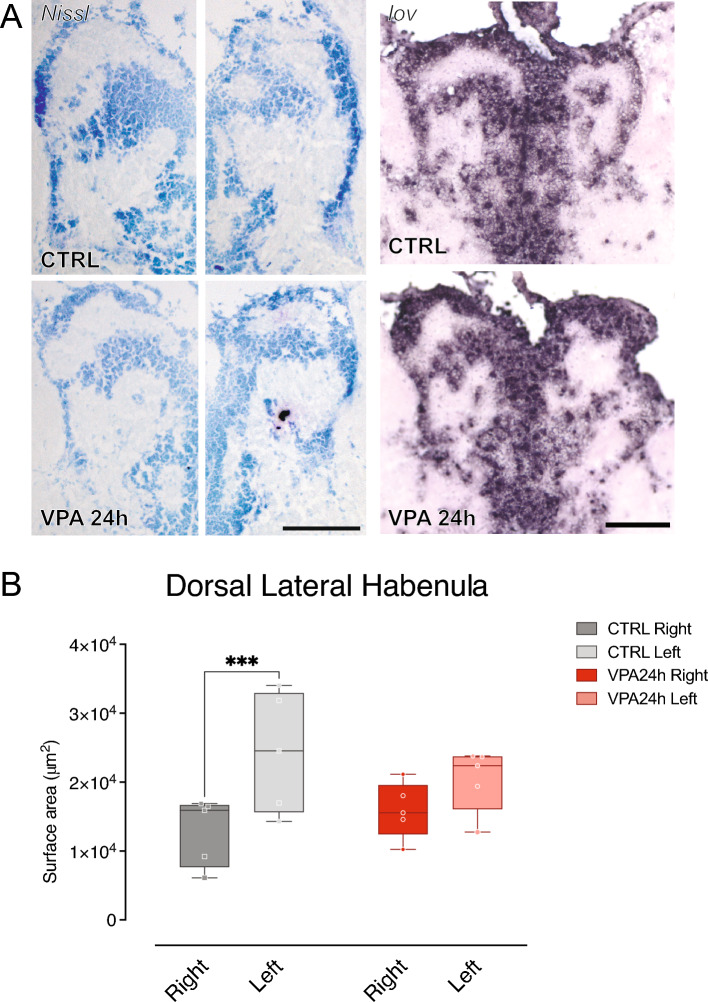
Neuroanatomical analyses of the dorsal habenual nuclei. (**A**) Top, representative images of Nissl-stained (left panel) and *in-situ* hydridisation of coronal sections in control (upper panels) and VPA-treated (lower panels) adult zebrafish habenulae. (**B**) Box and whisker plot (median, min to max) of surface area (µm^2^) quantification of Nissl-stained sections for each treatment group in the left and right habenular nuclei of adult zebrafish. ^***^p < 0.001. Scale bars 100 µm.

To assess the effect of VPA on the morphology and the size of the dorsal nucleus of the habenula, we performed morphometric analysis on Nissl-stained sections at the level of the habenula in adult zebrafish. Brain sections were prepared from five adult zebrafish from each treatment group, and surface area quantification was performed in all the sections that displayed an intact dorsal habenular nucleus. We measured the surface area of the nucleus in each section (1–2 for each animal) and performed repeated measure ANOVA, considering the difference between hemispheres and the effect of treatment. The analysis revealed a significant asymmetry in the morphology of the dorsolateral habenula that was different in the two treatment groups (Fig. 5B; interaction treatment * brain side F_(1,8)_ = 8.63512, *p =* 0.0188). In line with previous reports^43^, quantification of the surface area revealed a significantly larger area of the dorsolateral habenula of the left hemisphere compared to the right one in the control group, while the size of the two nuclei was not significantly different in the VPA treatment group (Fig. 5B; CTRL right–left *t*_(8)_ = 6.8434, *p =* 0.0005; VPA 24 h right–left: *t*_(8)_ = 2.6877, *p =* 0.1059; right CTRL–right VPA 24 h: *t*_(16)_ = 0.8041, *p =* 0.8967; left CTRL–left VPA 24 h: *t*_(16)_ = 1.0577, *p =* 0.7679), where the two sides appear to be symmetric.

To evaluate the effect of VPA treatment on the expression pattern of the epithalamic marker *kctd12.1/leftover*, we performed *in-situ* hybridization on brain sections of adult zebrafish. Visual inspection of the expression signal revealed a restricted expression of *kctd12.1/leftover* at this stage in the habenula of both control and VPA-treated animals. The expression pattern precisely delineates the difference between the left and the right domain in control animals, while in the VPA treatment group, no changes in the expression pattern of the gene are noticeable (Fig. 5A, panel on the right). In combination with the molecular data and the morphometric analysis, the assessment of the expression pattern of the epithalamic marker *kctd12.1/leftover* points to a *“symmetrization”* of the two sides of the dorsal habenular domain mediated by VPA exposure rather than to changes in the expression pattern of the marker *kctd12.1/leftover*.

## Discussion

Although cerebral asymmetry is recognized to be critical for cognitive development^37^ the role of brain lateralization in atypical social development is still unclear. Animal models are fundamental tools to investigate neurobiological mechanisms associated with human development and disorders, providing mechanistic insights into evolutionarily conserved functions. Here, we have investigated behavioral and biological lateralization in a zebrafish model of ASD based on the embryonic administration of VPA. A single embryonic exposure to VPA induces neuroanatomical and behavioral changes that resemble the core signs of ASD in many vertebrate species^52,53^. Previous studies in zebrafish have established a detrimental effect of VPA on social behavior^83,54,55^ at different dosages. A previous report^49^ has identified the lowest dose of VPA (1 µM) that minimizes the toxic effect on zebrafish embryo survival, maintaining its effect on the expression of several neurodevelopmental genes previously known to be affected by VPA^49,56,57^. However, the effect of lower doses of VPA on social behavior was not assessed. This study shows that one micromolar VPA dose not only induces social interaction deficits but also affects the left visual bias in a social recognition task, based on the animals’ response to their reflection in a mirror. Moreover, the gene expression data suggests that exposure to one micromolar VPA is sufficient to neutralize both the left visual bias and the asymmetric expression of *kctd12.1/leftover*. Given the importance of cerebral asymmetry for brain development and function, this work may open new perspectives on the study of brain lateralization and its link to typical and atypical social development in animal models.

Similar to the social affiliative responses shown by other vertebrate species towards visual cues in the face region^58–60^, zebrafish larvae exhibit spontaneous approach responses to their reflected image, reacting to the mirror image as to a social stimulus representing a conspecific^61,62^. Interestingly, zebrafish larvae observing their mirror image also display a stable left visual bias^38^ that is shared with other fish species^63–66^ and reaches a maximum at the same stage when social interaction begins to emerge (i.e. the third postnatal week^67^). This study analyzes the effect of VPA exposure on this social behavioral lateralization, revealing a dramatic effect of VPA on the left visual bias displayed by zebrafish larvae towards their reflected image. Both the 24- and 48-h VPA treatment groups showed severe impairment of the left visual bias, reducing their laterality index to chance levels, an indication of a *“symmetrization”* of the response to the social cues. VPA-treated zebrafish larvae thus fail to exhibit the typical lateralized response underlying the right hemisphere dominance for social recognition described in many vertebrate species, including humans (see for a review^37^).

The earliest sign of brain asymmetry in zebrafish is the activation of the Nodal pathway starting at about 18 hpf in the left epithalamus^68^. Shortly after the activation of the Nodal pathway, the parapineal appears on the left side of the pineal anlage and induces the left lateralization of the habenular nuclei^43,69^. The habenular nuclei show left–right differences in their size, their neuropil density and innervation pattern and the expression of the *kctd12.1/leftover* gene as early as 2 dpf^43^. Expression of the *kctd12.1/leftover* gene is originally asymmetric, being highly expressed on the (left) side closely opposed to the parapineal, and differently from other components of the Nodal pathway, continue to be highly expressed in the left epithalamus into adulthood^43^. Interestingly, in the small proportion of larvae with disrupted left–right asymmetry that develop bilateral parapineal organs, both habenular nuclei show high expression of the *kctd12.1/leftover* gene^43^. Wnt signaling also seems to be necessary for the communication between the parapineal organ and the left habenular nuclei to establish asymmetric activation of Nodal pathway genes in the brain^70^. Several studies have examined the functional relevance of loss or reversals of left epithalamic asymmetry induced by perturbation of habenular identity or disruption of the parapineal organ using genetic, pharmacological approaches or physical ablation in zebrafish^71–76^. The predominant view is that a reversal of the epithalamic asymmetry induces inverted or absent behavioral lateralization, with substantial differences underlying the different zebrafish strains, artificial selection, or genetic manipulation^72–75^. Our behavioral and molecular analyses demonstrate that embryonic treatment with VPA neutralizes the behavioral lateralization shown by the animal observing their reflected image and, at the same time, alters the expression of the gene *kctd12.1/leftover* (increasing its expression on the right side). Interestingly, our morphometric analysis of the dorsal habenula surface area, together with the expression pattern of the gene *kctd12.1/leftover,* suggest the *“symmetrization”* of the left and right habenular domains in VPA-treated animals. It remains to be established to what extent VPA acts on the molecular mechanisms driving brain asymmetry at early developmental stages and which of the molecular components are directly affected by VPA.

Notably, while this study provides clear evidence of an effect of VPA on behavioral lateralization of zebrafish at three weeks and of an altered expression of *kctd12.1/leftover* at three months of age, the data presented here are mostly correlative and do not allow to draw conclusions on a functional link between these two mechanisms. Future studies will clarify whether VPA directly affects the development of the epithalamus and if pharmacological or genetic manipulations of mechanisms of biological asymmetry could potentially restore functional lateralization in this context.

As for the lack of asymmetric expression in the other thalamic markers *kctd12.2/right-on* and *kctd8/dexter* in the control zebrafish, the neuroanatomical heterogeneous nature of the adult epithalamus could account for the differences compared to what previously shown during embryonic development. Evidence shows that *kctd12.2/right-on* and *kctd8/dexter* appear to have a differential regional distribution, such that *kctd12.2/right-on* and *kctd8/dexter* are expressed in the dorsal region of the right habenula (but not the left one) and in the ventral part of the left habenula^44^. Unlike most studies, the genes' asymmetric expression was assessed using a markedly quantitative technique that does not allow the differentiation of the neuroanatomical sub-regions. The lack of asymmetry we observed in our data might be due to the inability of the method to detect qualitative regional differences in the neuroanatomical distribution of these markers or to might be the results of differences in the genetic background or in the age of the animals. Previous studies have shown a lack of asymmetric *kctd12.2/right-on* and *kctd8/dexter* distribution. For example, Messina and collaborators^51^ did not report hemispheric differences in the expression of *kctd8/dexter* in adult (1-year-old) zebrafish of a commercial mixed strain. Similarly, Doll and colleagues^77^ reported a comparable number of cells expressing the gene *kctd12.2/right-on* in the two hemispheres of 4-dpf zebrafish of the AB strain.

In addition to brain asymmetry in the epithalamus, this study also analyzed the expression of pallial genes known to have a left–right asymmetric distribution in humans^78^, mice^79^ and zebrafish^51^. No asymmetric expression of the analyzed genes was detected in vehicle-treated zebrafish, while VPA treatment induced left lateralization of the expression of *arrb2*, *fez* and *nipa2*. As for the lack of correspondence between the left–right distribution of these pallial markers in control animals, one potential confound between the present study and the one from Messina et al.^51^, reporting asymmetrical expression of the same genes at the same stage, could be the inclusion in our samples of both pallial and subpallial regions. In addition, the expression of the pallial genes might also be influenced by the neuroanatomical distribution of defined cell subpopulation. Previous studies have already demonstrated an asymmetric distribution of GABAergic interneurons in the sensory cortex of mouse models for ASD^80^, including in animals exposed to VPA^81^.

This study shows, for the first time, that one micromolar dose of VPA is sufficient to induce social interaction deficits and to neutralize both the left visual bias in a social recognition task as well as the asymmetric expression of the epithalamic marker *kctd12.1/leftover*, suggesting new perspectives on the effect of VPA on brain development and proposing a new tool to investigate brain lateralization and its link to ASD in a zebrafish model.

## Materials and methods

### Ethical regulations

All husbandry and experimental procedures complied with the European Directive 2010/63/EU on the protection of animals used for scientific purposes and were approved by the Scientific Committee on Animal Health and Animal Welfare of our University and by the Ministry of Health (Protocol n. 333/2021-PR). The study is reported in accordance with ARRIVE guidelines (https://arriveguidelines.org).

### Animals

Adult AB wild-type zebrafish were moved into breeding tanks overnight, separated by a transparent barrier. The day after, the barrier was removed, and fish were left to breed. Embryos were collected in E3 medium (5.00 mM NaCl, 0.44 mM CaCl_2_, 0.33 mM MgSO_4_ and 0.17 mM KCl). At 5 hpf embryos were placed into 10 cm Petri dishes containing E3 medium (control) and E3 medium with 1 μM VPA (Sigma-Aldrich, P4543; Merck Life Science Srl, Milan, Italy) for 24 or 48 h. At the end of the treatment, the medium was replaced by E3 medium and zebrafish larvae were grown at 28.5 °C for three- or four-weeks post-fertilization before being subjected to behavioral studies (mirror test and social preference, respectively) or until adulthood (three months post-fertilization) before proceeding to brain microdissection.

### Mirror test

One hundred twenty-four larvae of the AB strain were observed at three weeks of age (Fig. 1A). Each experimental group was composed of 39 vehicle-treated controls (CTRL), 46 and 39 larvae treated with 1 µM VPA for 24 and 48 h, respectively. Each group was observed only once. The apparatus consisted of a circular amaranth tank (diameter × height: 175 × 27 cm), surrounded by a circular black curtain fixed on a wood-and-metal frame. The mirror test apparatus was placed inside the bigger tank and was composed of white plastic walls (20 × 5 × 8 cm), with mirrors on the long walls (one per side) and lit from above (height: 100 cm) through a 24-W fluorescent white light tube (Lumilux, Osram GmbH, D) (Fig. 1A). The water was 2.5 cm deep, and its temperature was maintained at 26 ± 1 °C using a 50-W heater (NEWA Therm®, NEWA). Each larva was placed in turn in the center of the test apparatus and video-recorded from above through a webcam (LifeCam Studio, Microsoft) for 5 min. The positions of each larva were manually scored offline every 2 seconds, by superimposition on the computer screen of a cursor on the long axis of the body, using the video recording. The body angle was taken relative to the closest of the two mirrors. All the positions where the larva was in a central 4 mm wide area were discarded. Positions in which the body was aligned parallel to the nearest mirror (“parallel observations”) and at an angle to the mirror (“angled observations”: 1°-179° towards the left or right eye use) were scored jointly.

### Social preference

Thirty larvae of the AB strain were examined at four weeks of age (Fig. 2A), each experimental group was composed of 12 controls (CTRL), 11 and 7 larvae treated with 1 µM VPA for 24 and 48 h, respectively. Each group was observed only once. The apparatus consisted of a circular amaranth tank (diameter × height: 175 × 27 cm), surrounded by a circular black curtain fixed on a wood-and-metal frame where the social preference apparatus was placed. The social preference apparatus was made of white plastic walls (11.2 × 4.2 × 8 cm) and divided into two chambers by a transparent barrier so that the experimental larva could see the companions. One of the chambers (the social chamber, 7 × 4.2 × 8 cm) hosted the experimental larva under test, while the other (4.2 × 4.2 × 8 cm) hosted 6 conspecifics (Fig. 2A). To analyze the social preference, the social chamber was divided into two zones (3.5 × 4.2 × 8 cm), one proximal to the conspecifics and one distal from them. The water was 2.5 cm deep and was kept at 26 ± 1 °C. The apparatus was lit from above (height: 30 cm) through a set of LED lights (~ 1700 lm). Each larva was placed in turn in the center of the social chamber and video-recorded using a high-resolution camera (FLIR Systems) for 5 min. The video recordings were coded offline, and the time spent by the larvae in the two zones was scored manually.

### Microdissection and RNA extraction

Three months-old AB zebrafish (Figs. 3A, 4A; six controls and six treated at 5 hpf with 1 µM VPA for 24 h) were anesthetized in an ice-cold water bath and sacrificed by decapitation; their brains were removed and dissected in ice-cold phosphate-buffered saline solution (PBS; Fisher Bioreagents, USA). The two hemispheres of the telencephalon (including the pallium and the subpallium) and of the thalamus were collected separately from each animal and used for total RNA extractions using the RNeasy Mini Kit (QIAGEN; Milan, Italy). Briefly, tissues were homogenized in lysis buffer, run onto RNeasy spin columns, treated with DNase (RNase-Free DNase Set, QIAGEN; Milan, Italy) and eluted in RNase/DNase-free water. Total RNAs were quantified using NanoDrop™ (Thermo Fisher Scientific; Monza, Italy), and reverse transcribed using the SuperScript VILO™ cDNA Synthesis Kit (Invitrogen, Thermo Fisher Scientific; Monza, Italy) according to the manufacturer's instructions.

### Quantitative real-time PCR

RT-qPCR experiments were performed using specific, commercially synthesized primer pairs (Merck Life Science Srl, Milan, Italy) as previously reported^49^. The triplicate reactions/samples were performed using the PowerUp™ SYBR™ Green Master Mix and a CFX96™ Real-Time System (Bio-Rad, Milan, Italy). The dCt method was used for expression quantification, raw expression data were normalized on the expression of the 18S reference gene. The lists of primers are reported in Table 5.

**Table 5:** List of primers used in the study.

Gene name	Gene ID	Primers	Sequence (5′-3′)	Amplicon size	Efficiency (%)
*18S ribosomal RNA*	100037361	For	TCGCTAGTTGGCATCGTTTATG	85 bp	93
		Rev	CGGAGGTTCGAAGACGATCA		
*kctd12.1/lov*	373865	For	AGTTCTTTCAGCTGCGGGACCTTA	112 bp	98.3
		Rev	GCGACGGACAGTGTGCGAGAG		
*kctd8/dex*	568933	For	CATGCCATCAATTACGCAAC	112 bp	101.9
		Rev	CGGATCCCGACTTTCATTTA		
*kctd12.2/ron*	553403	For	GCCACTCAACTTTGCTCTCC	191 bp	99.7
		Rev	GAGGCTCGCTTTCTCTTTCTCTTTGA		
*arrb2*	394099	For	CCCTTCAACTTCACGATTCC	127 bp	93
		Rev	CATCCACAGTTTTGGCACAG		
*fez1*	406705	For	AAACACTCAACGGCAACCTC	159 bp	95.2
		Rev	CCGGTGAGTTTTCCATCATC		
*gap43*	30608	For	GACACATCACCCGGAAAAAG	151 bp	96.4
		Rev	TCATTTGCTGGGGAGTTAGG		
*robo1*	30769	For	ATCTCAATCCCGAAGTGCTG	118 bp	97.5
		Rev	TTTCCTGCTCACAGACGATG		
*nipa1*	450042	For	AGTCTTGTTTGGTGCCGTTC	119 bp	101.4
		Rev	TGGGTGAGTGAATGATGAGC		
*nipa2*	406399	For	CTTTGTGGTGTTTGCGACAG	176 bp	98.5
		Rev	CAGCGATGGCCTCTTTAATC		

### Nissl staining and in-situ hybridization

Five adult zebrafish of each treatment group were sacrificed under anaesthesia by decapitation. Brains were rapidly removed and flash-frozen in dry ice. Cryostat 50- or 20 µm coronal sections were prepared for histological staining or *in-situ* hybridization, respectively. For Nissl staining, sections were fixed for 30 min in freshly prepared 4% paraformaldehyde (Carlo Erba Reagents) in phosphate buffered saline (PBS; pH 7.4). Brain sections were then briefly washed with distilled water and immersed into 1% cresyl violet for 30 min, washed in distilled water, dehydrated in an alcohol series, cleared in xylene, and coverslipped. For *in-situ* hybridization, 20-μm brain sections were fixed in 4% paraformaldehyde (Carlo Erba Reagents), rinsed in PBS, and hybridized with *kctd12.1/leftover* (*lov*) probes in a humidified chamber at 65º C overnight. Slides were washed in formamide/SSC solution (formamide: Invitrogen, ThermoFisher Scientific; 20 × SSC, saline-sodium citrate buffer: Gibco, ThermoFisher Scientific) at 65º C and in MAB solution (maleic acid buffer; Sigma-Aldrich/Merck) at room temperature. After incubation in blocking solution (10% Fetal Bovine Serum (Euroclone), blocking reagent (Roche) in MAB), the glass slides were incubated with an anti-DIG-AP antibody (Anti-Digoxigenin-AP, Fab fragments; Merck) overnight in a humidified chamber. Slides were treated with BCIP/NBT substrate of alkaline phosphatase (BCIP/NBT, Sigma-Aldrich/Merck) and kept in the dark until the colorimetric reaction reached the expected point. Finally, the slides were mounted using Fluoroshield with DAPI (Sigma-Aldrich/Merck). Stained sections were analyzed under a microscope (Observer.Z1, ZEISS) using a 20 × objective and a digital camera (Zeiss AxioCam MRc 5).

### Habenular morphometric analysis

Morphometric analysis was performed on Nissl-stained sections at the level of the habenula. Bright-field images of the habenular nuclei were acquired at 20× magnification and merged using Adobe Photoshop software. Brain areas were identified according to Aizawa et al.,2013^82^. The dorsolateral nucleus of the habenula was visually identified, and the area was manually outlined and the surface area quantified using the Fiji software (ImageJ2 version 2.14.0/1.54f., imagej.net).

### Statistical analysis

In the mirror test, differential eye use was evaluated using the visual index, calculated as the frequency of left-eye use/(frequency of right-eye use + frequency of left-eye use). Values significantly higher than 0.5 indicate a preference for left-eye use, while values significantly lower than 0.5 indicate a preference for right-eye use. To evaluate the social preference of zebrafish larvae, the following variables were measured: the percentage [%] of total time spent (TTS) in the proximal zone (referred to as social preference index^83^), the latency [s] to the first change of zone, and the number of alternations between the two zones (proximal-to-distal; distal-to-proximal) during the social preference test. Values of social preference index (%) were calculated as ((TTS in proximal zone/TTS proximal + TTS distal) × 100), the values range from 100% (full choice for the social (proximal) zone) to 0% (full choice for the distal zone), where 50% represents the absence of preference. The effect of treatment on the visual field index was evaluated by one-way analysis of variance (ANOVA), the effect of treatment and time on the social preference index, on the latency to the first change of zone and on the spontaneous alternations was evaluated by multifactorial ANOVA, surface area in the habenula was evaluated using repeated measure ANOVA. Statistical evaluation on the expression levels was performed on the log2 gene expression levels (dCt), the effect of treatment, brain side and transcript type were estimated using a linear mixed model, considering them as fixed factors and the experiment (experimental unit) as random factor. A Lateralization Index (LI) was calculated, using the linear expression levels in the two hemispheres, as: LI = (left expression–right expression)/(left expression + right expression). For all the tests, significant departures of the social preference index/ visual field index/LI from chance level (50%, 0.5 and 0, respectively) were estimated by one-sample two-tailed *t*-tests. Graphs were generated with GraphPad Prism 10, statistical analyses were performed with GraphPad Prism 10, using Šidák correction, or with Rstudio, using the *nlme* package (https://cran.r-project.org/web/packages/nlme/index.html) for the linear mixed models and the *emmeans* (https://cran.r-project.org/web/packages/emmeans/index.html) package for Tukey pairwise comparison tests. Alpha was set to 0.05 for all tests.

### Ethical approval

This research was conducted in the Animal Cognition and Neuroscience Laboratory (ACN Lab) of Center for Mind/Brain Sciences (CIMeC, University of Trento, Italy). All husbandry and experimental procedures complied with European Legislation for the Protection of Animals used for Scientific Purposes (Directive 2010/63/EU) and were reviewed and authorized by the University of Trento’s Ethic Committee for the Experiments on Living Organisms and by the Italian Ministry of Health (auth. no. 333/2021-PR issued pursuant to art. 31 of Legislative Decree 26/2014).

### Supplementary Information

Supplementary Information.

## Data Availability

The datasets generated and analyzed during the current study are included in this published article as supplementary information file.
